# In Situ Observation of ZnO Nanoparticle Formation by a Combination of Time-Resolved X-ray Absorption Spectroscopy and X-ray Diffraction

**DOI:** 10.3390/ma15228186

**Published:** 2022-11-18

**Authors:** Franz Eckelt, Patrick Rothweiler, Frederic Braun, Lukas Voss, Ankica Šarić, Martina Vrankić, Dirk Lützenkirchen-Hecht

**Affiliations:** 1 Faculty of Natural Sciences, University of Wuppertal, Gauss-Str. 20, 42119 Wuppertal, Germany; 2 Centre of Excellence for Advanced Materials and Sensing Devices, Division of Materials Physics, Ruđer Bošković Institute, HR-10002 Zagreb, Croatia

**Keywords:** Zn-oxide synthesis, in situ investigation, solution chemistry, X-ray absorption spectroscopy, X-ray diffraction

## Abstract

The formation of ZnO nanomaterials from different Zn acetylacetonate precursor solutions was studied in situ by employing simultaneous, time-resolved X-ray diffraction (XRD) and X-ray absorption spectroscopy (EXAFS) at the Zn K-edge. The precursor solutions were heated from room temperature to the desired reaction temperatures in a hermetically sealed cell dedicated to X-ray experiments. In general, the first indications for the formation of hexagonal ZnO were found for elevated temperatures of about 80 °C both by XRD and EXAFS, and the contributions increase with temperature and time. However, no reaction intermediates could be proved in addition to the Zn precursors and the formed hexagonal ZnO materials. Furthermore, the results show that the efficiency of the reaction, i.e., the conversion of the precursor material to the ZnO product, strongly depends on the solvent used and the reaction temperature. ZnO formation is accelerated by an increased temperature of 165 °C and the use of 1-octanol, with a conversion to ZnO of more than 80% after only a ca. 35 min reaction time according to a detailed analysis of the EXAFS data. For comparison, an identical concentration of Zn acetylacetonate in water or dilute alkaline NaOH solutions and a reaction temperature of around 90 °C leads to a smaller conversion of approximately 50% only, even after several hours of reaction. The particle size determined from XRD for different orientations shows a preferred orientation along the c-direction of the hexagonal crystal system, as well in accordance with scanning electron microscopy. The LaMer model explained this highly non-uniform growth of needle-like ZnO crystallites.

## 1. Introduction

Zinc oxide (ZnO) has many interesting applications in various fields, such as catalysis, energy harvesting and storage, electronics, sensors, biomedicine and cosmetics, to mention just a few. With the advances in nanotechnology, new opportunities for preparing ZnO nanoparticles with tailored morphology and physicochemical properties appeared. In general, the preparation of ZnO nanoparticles from solution-based processes appears advantageous due to a high level of controllability and the opportunity to scale the reaction to a technological scale. In particular, hydrothermal synthesis routes for ZnO employing Zn-containing precursors in aqueous solutions became popular due to their simplicity and variability [1,2]. Important parameters for the reaction are the concentration of the Zn-precursor, possible additives and the reaction temperature leading to entirely different morphologies of the ZnO particles [2]. However, as the reaction temperature is limited by the water solvent′s boiling point (T_B_), different solvents with increased T_B_ appear attractive. In particular, organic solvents thus have gained interest for such a solvothermal synthesis [3]. Due to the use of different additives and the variation of the solution pH, temperature, pressure and time, these strategies are well-suited to synthesize a broad range of ZnO nanomaterials with different micro- and nanostructures and morphologies, ranging from micro- and nanospheres [3,4], nanoparticles, nanoflowers, and nanopyramids [3,5,6] to needle-like deposits with hollow structures [7]. The influence of an alcoholic solvent on the ZnO particle growth and morphology has been proved experimentally and theoretically [6,8,9,10,11,12], and microwave-assisted growth has also been demonstrated [3,13,14]. A comprehensive review of different preparation strategies has recently appeared [15].

Though studies focusing on the influence of synthesis conditions on structure and morphology have made progress, there is still a long way to go before a comprehensive understanding of the effects of synthesis parameters on structure, particle size, and morphology can be obtained. Consequently, the tailored preparation of ZnO nanomaterials with optimized structures and morphologies for specific purposes is still challenging. An in situ analysis and control of ZnO particle formation processes would thus be valuable and helpful for a target-oriented growth of nanomaterials. Specifically, the investigation of the in situ processes of ZnO formation in the liquid phase and at elevated temperatures continue to face obstacles that must be overcome.

Therefore, it is necessary to use analytical techniques compatible with the harsh reaction environment (such as solvents, temperatures, pressures, reaction speed, etc.) Electron-based techniques, such as electron microscopies (scanning electron microscopy, SEM; transmission electron microscopy, TEM) and electron spectroscopies (e.g., X-ray or ultraviolet photoelectron spectroscopies, XPS and UPS) that can substantially contribute to the elucidation of the structure, the morphology and the electronic properties of nanomaterials are not suited for in situ investigations in the liquid phase. In particular, functional groups of the species in the liquid phase can be identified using a visible, microwave, or infrared spectroscopy, such as UV–Vis, IR, or Raman spectroscopy (see, e.g., [16,17]). If the concentration of the species of interest—such as, e.g., small ZnO nuclei in the present case—is small compared to, e.g., the solvent or additives in the reaction solution, then the quantitative results are challenging to obtain. Furthermore, in conventional IR spectroscopy, the capability to characterize nanoparticle surfaces and their chemistry is limited. Nevertheless, although a quantitative identification of hydrolyzed or condensed species is impossible to obtain even from a thorough UV–Vis spectroscopy analysis, the kinetics of the nanoparticle formation could be derived, i.e., the time domain for hydrolysis, condensation and aggregation of primary particles could be obtained [16,18].

In this context, the use and combination of X-ray-based, photon-in, and photon-out techniques, such as X-ray scattering [19] and X-ray absorption spectroscopy [20] appear favorable. While X-ray scattering probes the sample structure at a long range, providing detailed information about crystalline structures, their size and orientation [19], X-ray absorption spectroscopy techniques (EXAFS and XANES) are sensitive to the short-range order around the X-ray absorbing atom, its chemical valence and the bond geometry [20]. Thus, EXAFS and XANES are well-suited for investigating amorphous materials, molecular complexes, and liquids [20,21]. For the study mentioned above, EXAFS and XANES measurements recorded simultaneously with the UV–Vis data allowed for the identification of the titanium (Ti^4+^) tetraisopropoxide precursor as well as intermediate dodecatitanate condensate species within a Ti_11_O_13_ or Ti_12_O_16_ molecular framework, and amorphous TiO_2_ particles in later stages of the growth [18]. Depending on the temperature and the hydrolysis rate, the kinetics of the reactions substantially changed, as already indicated by the UV–Vis data [16,18].

Koziej et al. recently reported on the in situ investigation of the formation of spherical CoO nanocrystals from metal-organic precursors Co(acac)_3_ in an alcoholic solution when applying temperatures of 150 °C and above [22]. EXAFS/XANES revealed the rapid reduction from the pristine Co^3+^ (Co(acac)_3_) species to Co^2+^ in the form of Co(acac)_2_ within a few minutes, followed by the formation of small CoO oxide nuclei. A deconvolution/linear combination fit allows for the determination of Co(acac)_3_, Co(acac)_2_ and CoO concentrations as a function of time, with decreasing contributions of Co(acac)_3_, an intermediate Co(acac)_2_ species and conversion to about 90% of CoO in later stages of the reaction after ca. 60 min at about 160 °C [22]. These examples exemplarily indicate the useful combination of different X-ray techniques for the in situ, time-resolved analysis of nanoparticle formation processes.

In the case of ZnO, numerous ex situ X-ray studies successfully dealt with the characterization of differently prepared ZnO nanomaterials. The structure of ZnO at ambient conditions adopts the space group symmetry P6_3_*mc* (i.e., the Wurtzite structure, *W*). For moderate pressures, this structure can be transformed into a cubic setting within an F-43*m* space group (i.e., rock salt structure, *RS*) [23]. In contrast, the growth of a third structural modification of ZnO, namely the cubic zincblende type ZnO, is still challenging [24,25]. Compared to the bulk oxide, Wurtzite ZnO nanorods prepared by oxidation of metallic Zn in NaCl solutions exhibited more disorder and slightly larger Zn-Zn interlayer distances as investigated by EXAFS measurements [26]. Wurtzite nanoparticles with a size of about 3–5 nm prepared by hydrolysis from Zn(acac)_2_ under ultrasonic excitation with organic additives revealed a contraction of the Zn-O bond according to the strong interaction of Zn and oxygen [27]. 

At first glance, one may think these minor structural differences are negligible; however, the electronic structure and resulting physicochemical properties sensitively rely on these structural details. For example, slightly reduced ZnO_x_ nanomaterials reveal an improved catalytic reactivity and a good selectivity for the propane dehydrogenation reaction [28]. The antibacterial properties of ZnO nanostructures depend on the growth conditions and the size and orientation of the particles on the substrate [29]. Similarly, the photocatalytic reduction of CO_2_ by ZnO microspheres, microflowers and nanorods sensitively depends on the percentage of exposed [0001] facets and the size of the catalysts [30]. The EXAFS experiments revealed detectable differences in the performance of hollow tubular ZnO nanostructures for the photocatalytic degradation of organic dye pollutants [11], while XANES showed the ability to distinguish coordination changes and surface disorder of the Zn-O adsorption complexes [31].

The performance of hollow tubular ZnO nanostructures for the photocatalytic degradation of organic dye pollutants can be sensitively monitored by EXAFS experiments that reveal detectable differences [11]. Moreover, XANES has proven to differentiate the coordination changes and surface disorder of the Zn-O adsorption complexes [31]. Since the use of X-ray absorption spectroscopy at the Zn K-edge allows the coordination changes and surface specificities of ZnO-based nanostructures to be distinguished, the application of this technique with a complementary XRD approach is an efficient and unique method for the *immediate* monitoring of the in situ synthesis of ZnO nanomaterials during the processing, providing relevant results on structural and microstructural features essential for the definition of the optimal ZnO nanostructures, which have applications across a variety of fields, as illustrated in many studies ([5,11,15,28,29,30] and references therein).

In this contribution, we, therefore, demonstrate the simultaneous application of X-ray diffraction and X-ray absorption spectroscopy to follow the formation of ZnO nanoparticles from a metal-organic (Zn(acac)_2_) precursor in different solutions in situ with a time resolution of 2 s for each spectrum/diffractogram, so a very detailed analysis of the ZnO nanomaterials formation dynamics is feasible. A dedicated cell suited to a chemical synthesis employing inflammable liquids at temperatures up to ca. 165 °C and simultaneous X-ray measurements were conducted and realized for the experiments.

## 2. Materials and Methods

### 2.1. ZnO Synthesis Procedures

We employed Zinc acetylacetonate monohydrate (Zn(acac)_2_, Zn(C_5_H_7_O_2_)_2_H_2_O, Alfa Aesar, Haverhill, MA, USA), sodium hydroxide (NaOH, Sigma-Aldrich, St. Louis, MO, USA), 1-octanol (CH_3_(CH_2_)_7_OH, Merck Millipore, Burlington, MA, USA) and triple distilled water as starting materials for the synthesis of ZnO nanomaterials. The reactants were mixed at room temperature in desired amounts and stirred for about 10 min. Subsequently, the solution was poured into the reaction cell and carefully sealed. Finally, the precursor solution was heated to the desired temperature under continuous stirring. The sample notation and the experimental conditions used for the preparation of the selected samples are given in Table 1. In a typical synthetic procedure, zinc acetylacetonate monohydrate (Zn(acac)_2_) in the desired amount was mixed with water, a well-defined volume of aqueous NaOH solution (10^−3^ M) or 1-octanol, respectively, following the procedures described by Šarić et al. [4,7]. Depending on the actual solvent, the solutions were heated to about 90 °C (samples A and W) and 165 °C (sample O) to initiate the formation of ZnO nanomaterials for different reaction times, as compiled in Table 1. More than 25 individual preparations were performed and analyzed to ensure the reproducibility of the experiments.

### 2.2. In Situ Reaction Cell

**Figure 1:**
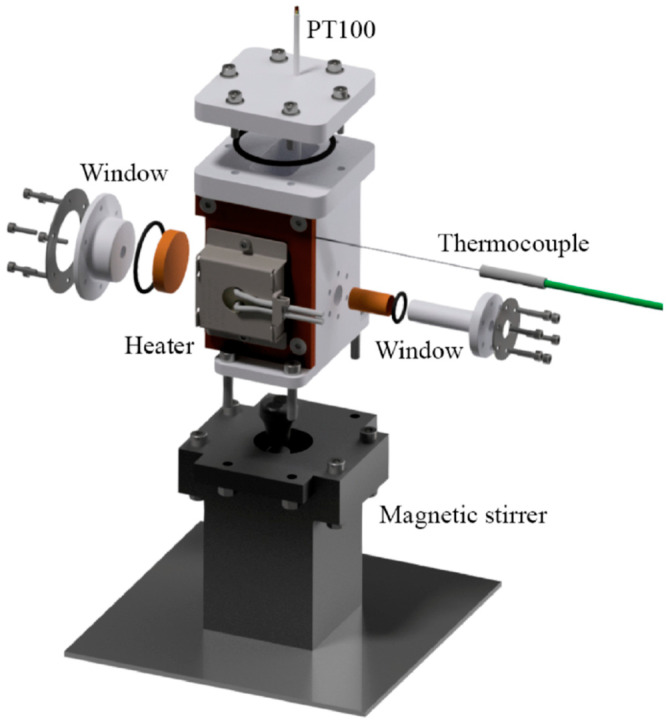
Schematic drawing of the reaction cell for hydrothermal/solvothermal preparation of ZnO nanomaterials. The X-ray beam passes from the right to the left.

The reactions were performed in a dedicated, hermetically sealed reaction cell, as shown in Figure 1. The main body of the cell with outer dimensions of 53 mm (width), 48 mm (length in the direction of the X-ray beam) and 102 mm (height) consists of a PTFE container in which the liquid precursor solution (maximum ca. 70 mL) is filled. Tests have shown that the electric heater attached to one side of the cell can heat the liquid sample solutions to a maximum temperature of ca. 300 °C, as measured with a platinum resistance thermometer encased in PTFE directly in the solution, and a second thermocouple fixed to the heater with a precision of ± 1 °C. The X-ray beam passes through the liquid sample volume about 20 mm above the bottom of the cell. It enters the cell via a small circular polyimide (Kapton) entrance window of 10 mm diameter and 25 μm thickness. The entrance and exit windows were mounted on specially designed PTFE frames employing a temperature and chemical-resistant adhesive (CMC 70110, CMC Klebetechnik, Frankenthal, Germany). It is important to note that the exit-window frame has a conical shape to allow scattering angles of up to 40°. The frames with the windows, as well as the top flange of the cell, are screwed to the main body and sealed by Viton O-rings of a suited diameter (see Figure 1). Moreover, the effective thickness of the probed sample can be varied by varying the length of the window frames so that a reasonable X-ray absorption is achieved on the one hand while the diffracted volume is kept small enough to reduce the broadening of the Bragg peaks on the other. To guarantee a homogenous temperature distribution within the cell and to avoid precipitation of reaction products at the cell walls and the windows, a magnetic stirrer actuated by a brushless motor in the cell holder below the liquid were employed. The stirrer, as well as the control of the heater and the temperature readouts, were remote-controlled via a Raspberry Pi with an Ethernet connection.

### 2.3. X-ray Experiments

**Figure 2:**
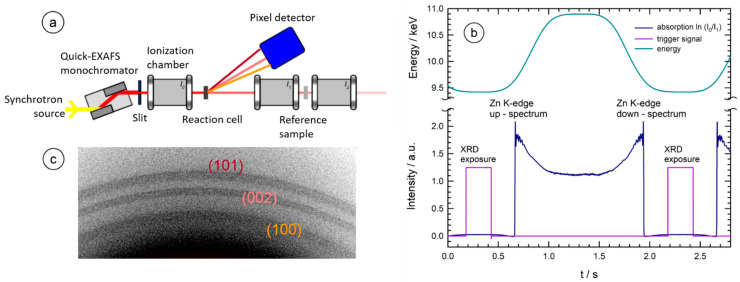
(**a**) Schematic representation of the experimental setup with the fast-scanning monochromator, the ionization chambers and the flat-panel detector arranged in forward-scattering geometry from the sample. (**b**) Continuous variation of the monochromator energy as a function of time, together with the measured absorption spectrum of the sample, and the trigger signal placed in the flat pre-edge region of the measurement. (**c**) Example of a diffraction pattern collected during the formation of ZnO nanoparticles in Zn(acac)_2_ solution in 10^−3^M NaOH (sample A) at a temperature of ca. 85 °C (t = 3200 s) within 250 ms of integration time. The (100)-reflection with a Bragg angle Θ_B_ of Θ_B_ ≈ 27.0° is located at the bottom of the active area of the pixel detector, the (002) with Θ_B_ ≈ 29.3° in the center, and the (101) in the upper region with Θ_B_ ≈ 30.8°, respectively, as also indicated by the colors in (**a**).

The X-ray experiments were carried out at the advanced spectroscopy beamline P64 at the PETRA III storage ring (DESY, Hamburg, Germany). A cryo-cooled, Si(111) channel-cut monochromator suited for fast oscillations with up to 100 Hz (i.e., up to 200 spectra per second) was employed for the EXAFS measurements [32]. Gas-filled ionization chambers were used to monitor the incident (I_0_) and transmitted (I_1_) X-ray intensities. A third ionization chamber (I_2_) was used to simultaneously investigate a Zn-metal reference foil with each sample spectrum for precise and reliable energy calibration, as depicted in Figure 2a. A Pilatus 100K pixel detector (DECTRIS, Dübendorf, Switzerland) was used to process the XRD images with forward-scattering geometry. While a sinusoidal excitation of the monochromator oscillation is usually employed for quick-scanning EXAFS experiments at P64 [32], it is important to perform X-ray diffraction studies with well-defined X-ray energy to obtain high-quality diffraction data. Thus, to avoid an interruption in the EXAFS data collection by stopping the oscillatory monochromator movements, we modified the sinusoidal movements by adding a signal of the third harmonic wave with suited amplitude so that the resulting oscillation obeys an energy plateau at the extremal points as can be seen in Figure 2b. With a trigger placed in this plateau by the monochromator control software, the illumination of the XRD detector can be initiated for a chosen period of time [33]. For the present experiments, a scan speed of 0.5 Hz was chosen, i.e., an EXAFS spectrum at the Zn K-edge (9.659 keV) covering the range from ca. 9.418 keV to 10.897 keV was taken every second, and an exposure of the XRD-detector was triggered every 2 s with an exposure time of 250 ms. It is important to note that the energy spread during the illumination of the XRD detector amounts to only 6.5 eV within this configuration, allowing for high-resolution XRD data to be obtained, as shown in Figure 2c. It should be noted that the used Pilatus detector has a rectangular active area of ca. 83.8 × 33.5 mm^2^, so that in an upright position, a larger Bragg-angle range can be covered in comparison to a horizontally orientated detector. However, for this study, we chose the latter position because it allowed us to detect a large portion of the diffraction cones at the most prominent (100), (002), and (101) diffraction peaks of hexagonal ZnO, enabling the detection of subtle structural changes in the diffracting nanoparticles (see, e.g., refs. [4,11,12]).

Complementary, static in situ and ex situ X-ray experiments were performed at beamline 10 of the DELTA storage ring (Dortmund, Germany) [34]. Here, the reference spectra of Zn metal, polycrystalline Wurtzite ZnO powder, and zinc acetylacetonate monohydrate (Zn(acac)_2_, Zn(C_5_H_7_O_2_)_2_ H_2_O) were recorded for comparison and their use in the linear combination fitting of the EXAFS data. EXAFS data reduction and analysis were performed using the Athena/Artemis software [35].

## 3. Results and Discussion

### 3.1. X-ray Absorption Spectroscopy

**Figure 3:**
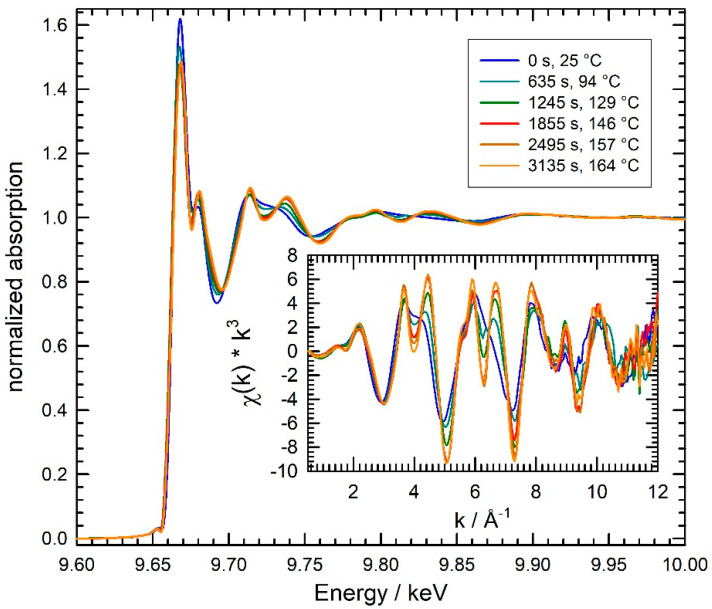
Selection of characteristic Zn K-edge X-ray absorption data measured during the heating of a Zn(acac)_2_ solution in 1-octanol from room temperature to ca. 165 °C as indicated (sample O). The k^3^-weighted EXAFS χ(k)*k^3^ extracted from the data is depicted in the inset.

Combined, simultaneous quick-EXAFS at the Zn K-edge and XRD measurements were measured during the synthesis of ZnO nanomaterials from different precursor solutions and for different temperatures and concentrations of the Zn(acac)_2_ precursors. As a typical example of quick-EXAFS data obtained during such a reaction, some selected spectra measured during the formation of ZnO in a solution containing 1-octanol as solvent (sample O, see Table 1) are presented in Figure 3. Although the spectra were collected with an acquisition time of only 1 s, the data were of sufficient quality to allow the extraction of k^3^-weighted absorption fine structure oscillations χ(k)*k^3^ up to a photoelectron wave vector of 11 Å^−1^, as shown in the inset of Figure 3. As can be seen, substantial changes in the spectra were observed when the stirred precursor solution was heated to about 100 °C and more. First, no changes in the absorption edge position defined as the inflection point of the spectra are observed. Thus, the chemical valence of the absorbing Zn-atoms does not change during the reaction, and the observed values of E_Edge_ = 9.663 keV ± 0.5 eV are typical for organic and inorganic Zn^2+^ species as expected [36,37]. While the white line intensity directly at the edge (9.67 keV) continuously decreases with increasing temperature, the absorption minimum at about 9.69 keV increases and slightly shifts to higher energies in the course of the reaction, and more prominent changes are observed for higher energies.

**Figure 4:**
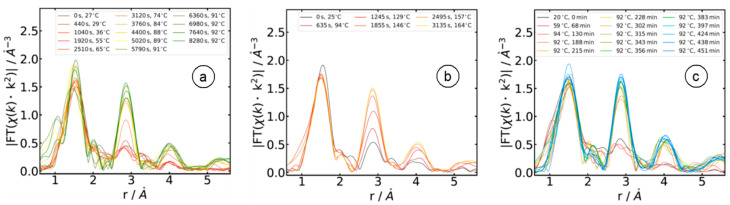
Magnitude of the k^2^-weighted X-ray absorption fine structures χ(k)*k^2^ for the indicated times and temperatures (k-range for the FTs: 1.2 Å^−1^ < k < 11.0 Å^−1^, Kaiser–Bessel window functions). The peaks at ca. 1.5 Å belong to the Zn-O interactions, the peaks at ca. 2.9 Å are due to Zn-Zn bonds, respectively, and peaks at ca. 4 Å belong to multiple scattering events with larger path distances involving zinc and oxygen backscatters. (**a**) The reaction of Zn(acac)_2_ solution in 10^−3^ M NaOH (sample A); (**b**) reaction of Zn(acac)_2_ solution in 1-octanol (sample O); and (**c**) reaction of Zn(acac)_2_ solution in distilled water (sample W).

Most notable is the presence of isosbestic points in the absorption spectra at ca. 9.677 keV, 9.711 keV, 9.732 keV, 9.752 keV and 9.822 keV. In the χ(k)*k^3^ data (inset of Figure 3), these characteristic features are visible at 4.2 Å^−1^, 5.6 Å^−1^, 6.4 Å^−1^, 7.1 Å^−1^ and 9.2 Å^−1^, underlining the presence of only two different compounds in substantial concentration in the sample volume, i.e., on first glance, we cannot give any evidence for reaction intermediates as, e.g., proposed by calculations in reference [11]. The reaction course can be more sensitively followed in the Fourier-transformed X-ray absorption fine structure data, as displayed in Figure 4 for the three different reaction media. Peaks in these Fourier transforms correspond to the first few coordination shells around the X-ray absorbing Zn atom, i.e., the first peak at ca. 1.5 Å radial distance corresponds to the first Zn-O coordination of the reaction educts and products, while the peaks at a larger space are related to the Zn-Zn coordinations and multiple scattering events in the structures present at a particular time. It should be noted that the peaks in the Fourier transforms are generally shifted to smaller distances by about 0.4 Å compared to their crystallographic positions due to the phase shifts of the scattered photoelectrons causing these peaks [20]. For example, the first Zn-O and Zn-Zn bond distances in Wurtzite are 1.97 Å and 3.24 Å, respectively [11,38], while the related peaks in the Fourier transform appear at 1.5 Å and 2.9 Å.

As can be seen in Figure 4, the position and the intensity of the peak at 1.5 Å remained almost constant as a function of the reaction time for all three preparations, while substantial differences were observed for the peaks at 2.9 Å and 4.0 Å, where a time- and temperature-dependent increase in intensity were observed. Comparing the Fourier-transform of Zn(acac)_2_ and crystalline ZnO reference samples, it can be concluded that the peak at 1.5 Å arises from the Zn-O bonds in both compounds. Since Zn is 4-coordinated with oxygen in both sample systems and the Zn-O bond distances are very similar in both cases, there is not much change expected in the first peak during ZnO particle formation. The slight decrease in intensity, which can be observed in all samples, resulted from the increasing temperature and the resulting increase in thermal vibrations so that the amplitude of the EXAFS fine structures χ(k)*k^2^ and their Fourier-transform are reduced accordingly. The peak at 2.9 Å arises from the Zn-Zn shell in crystalline ZnO, which has a coordination number of 12 in hexagonal ZnO at a distance of 3.24 Å. Since this shell is not present in Zn(acac)_2_, it forms only during the synthesis and is a consequence of the solvothermal formation of ZnO, as is the third peak at 4.0 Å, which is due to multiple scattering of the Zn-O-Zn pathways [11]. Therefore, the course of the ZnO reaction can be easily followed by examining these peaks. Obviously, the reaction kinetics of sample O (reaction in 1-octanol) are much faster compared to sample A (10^−3^ M NaOH), and especially to sample W (pure water).

For a more detailed analysis, the grown ZnO nanomaterials were extracted from the reaction cell after the completion of the reaction, filtered on paper, dried in air and investigated with EXAFS in transmission subsequently. The obtained experimental EXAFS data were fitted to a structural model based on the assumption of a hexagonal ZnO Wurtzite structure adopting a P6_3_*mc* space group symmetry, with unit cell metrics of *a* = *b* = 3.251 Å, *c* = 5.207Å, *α* = *β* = 90° and *γ* = 120° [38]. All atoms up to a radius of 4.7 Å to the central Zn atom were considered for calculating the phase and amplitude functions using the FEFF 9 code [39], resulting in 14 coordination shells and a cluster size of 39 atoms. 

Each coordination shell (i) considered is characterized by its distance (R_i_), the number of atoms in the shell (N_i_), the inner potential shift (E_0,i_), the mean square displacement (σ_i_^2^) and the amplitude reduction factor (S_0,i_^2^). If each shell considered had been fitted with its own set of parameters, the results here would be a very large set of parameters with a statistically non-significant number of variables. The significance limit for the number of fit variables (N_ipd_) can be estimated from the energy range measured during the experiment, the k-range used for the Fourier transform (here, the range from k_min_ = 1.2 Å^−1^ to k_max_ = 12.4 Å^−1^), and the R-range in the FT used for the fit (here, R_min_ = 1.0 Å to R_max_ = 4.3 Å); for details see, e.g., ref. [40].

**Figure 5:**
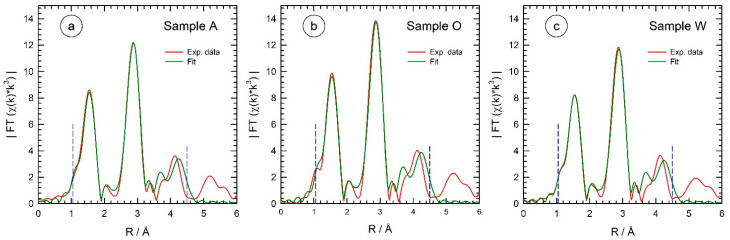
Fits of the EXAFS data measured after extraction of the prepared ZnO nanomaterials from the reaction cell after finalization of the reaction. The data were fitted to a model structure consisting of hexagonal, wurtzite-type ZnO (k-range for the fits: 1.2 Å^−1^ < k < 12.4 Å^−1^, Kaiser-Bessel-window functions, R-range 1.0 Å < R < 4.3 Å, dashed vertical blue lines). (**a**) The reaction of Zn(acac)_2_ solution in 10^−3^ M NaOH (sample A); (**b**) reaction of Zn(acac)_2_ solution in 1-octanol (sample O); and (**c**) reaction of Zn(acac)_2_ solution in distilled water (sample W).

For the present experiments, N_idp_ had a value of N_idp_ ≈ 23. To ensure this upper level of parameters was not exceeded, a simplified fit model was developed, which assumes identical values for S_0_^2^ and σ_i_^2^ on all the Zn-O shells. Similarly, the Zn-Zn scattering was described by a separate set of parameters. The distances R_i_ were varied individually for the first single scattering of Zn-O (R_1_) and the Zn-Zn shells (R_2_) to sensitively model small structural differences. All other distances were fitted with a single scaling factor (α) of the original ZnO Wurtzite lattice (R_eff_), so the fitted distances for all paths with longer bond distances were given by R_eff_*α. To minimize the influence of the correlation of the potential shift and all distances, the same value for E_0_ was used for all shells. Thus, this model has only eight fit variables compared to the significance limit (N_idp_ ≈ 23) and accordingly, the results are likely to have a high statistical significance. The fits were performed in R-space employing the k^3^-weighted absorption fine structure data using Artemis software, and the fit results are compiled in Figure 5 and Table 2, where the R-factors of the fits are also shown.

The fit results show excellent agreement with the Wurtzite structure for all samples studied, regardless of the details of the preparations, i.e., all paths lead to Wurtzite-type nanomaterials, in good agreement with the literature. There are only minor differences in the extracted short-range order structures; for example, in an overall trend, the distances are larger for sample A compared to samples O and W, while the intensity of the features (S_0_^2^) is maximal for sample O, i.e., the preparation in 1-octanol. For the disorder, no distinct differences are detectable, in agreement with recent EXAFS measurements [11].

**Figure 6:**
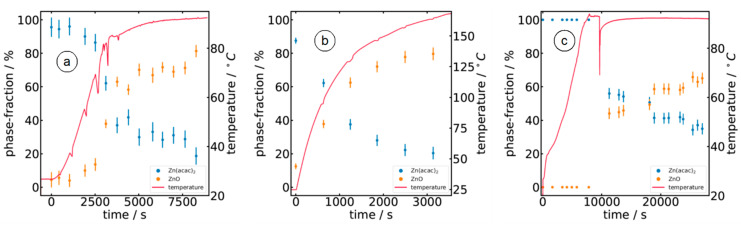
Results of linear combination fits of the EXAFS data measured in the course of the reaction in (**a**) Zn(acac)_2_ solution in 10^−3^ M NaOH (sample A); (**b**) Zn(acac)_2_ solution in 1-octanol (sample O); and (**c**) Zn(acac)_2_ solution in distilled water (sample W), respectively. Reference spectra of crystalline ZnO and Zn(acac)_2_ were used for the fits.

Since the reaction products in all cases are nanocrystalline ZnO materials in a Wurtzite structure, and due to the fact that the isosbestic points observed in the raw data and in the extracted EXAFS functions (χ(k)*k^3^, see Figure 3), it is very likely that only two phases are present during the reaction, namely the Wurtzite ZnO and the Zn(acac)_2_ precursor. Therefore, we performed linear combination fitting of the measured EXAFS data during the three preparation routes investigated here. The Zn K-edge spectra of the respective reference materials at room temperature were taken as constituting components, although the temperature during the reaction is substantially increased to about 90 °C for the reaction in 10^−3^ M NaOH (sample A) and distilled water (sample W) and to more than 160 °C for the reaction in 1-octanol (sample O). The fit results obtained for the different preparations are compiled in Figure 6 as a function of time, together with the measured temperature profiles of the reaction media.

There are clear differences depending on the solvent used. Namely, in the NaOH sample (sample A), the ZnO formation process starts after about 2000 s and a temperature of 55 °C. Up to about 5000 s and a temperature of about 90 °C, the ZnO phase fraction has increased to about 70% and remains at about this value until the end of the synthesis process considered. For sample A, the conversion process from Zn(acac)_2_ to ZnO seems to be completed after about 5000 s and at a temperature of 90 °C.

A similar formation process can be observed with water as the solvent (sample W). It took about 120 min (ca. 7000 s) to detect the first evidence of the presence of ZnO, again at a temperature of ca. 90 °C. After about 200 min, i.e., another 3000 s at a temperature of 90 °C, the ZnO content increases to 45% and reaches about 65% at the end of the observation period after a total of 450 min. Here, too, the Zn(acac)_2_ precursor has not yet been completely converted into ZnO after the period under consideration.

For the reaction in 1-octanol as solvent (sample O), ZnO is directly formed even at room temperature, i.e., ZnO is detectable at the initiation of the measurements with a fraction of ca. 10%. This proportion increases continuously with time and temperature and amounts to 35% after 600 s (90 °C), 60% after 1200 s (125 °C) and ca. 80% at the end of the observed process after 3200 s and a temperature of 165 °C. Thus, the opportunity to increase the reaction temperature to substantially more than 100 °C in the organic solvent increases the reaction rate and the conversion efficiency (i.e., the degree of transformation from Zn(acac)_2_ to ZnO) of the process. It should be noted that similar results were found for the formation of ZnO nanocrystals by adding KOH solution to an ethanolic zinc oxy-acetate solution at a slightly elevated temperature of 40 °C by parallel X-ray absorption spectroscopy, small angle X-ray scattering, and UV–Vis spectroscopy [41]. Similarly, the conversion efficiency of such a hydrolysis-condensation process after a reaction time of several hours reaches about 80% [41]. The increase in reaction temperature obviously helps to overcome the activation energy of the reaction due to the rise of the thermal kinetic energies of the reactants, and it further increases the number of collisions between the reactants, leading to substantially larger reaction rates.

### 3.2. X-ray Diffraction

**Figure 7:**
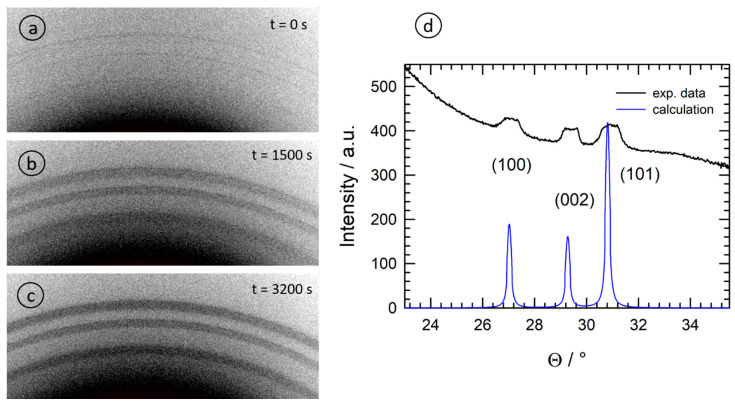
Selected diffraction patterns collected during the solvothermal reaction of Zn(acac)_2_ in 1-octanol (sample O) for a photon energy of 9.423 keV; (**a**) t = 0 s; (**b**) t = 1500 s; (**c**) t = 3200 s. In (**d**), the diffractogram calculated by azimuthal integration is compared to a theoretical calculation for ZnO having Wurtzite structure without any preferential orientation (space group P_63_mc, a = b = 3.251 Å, c = 5.207 Å; α = β = 90°, γ = 120°, [38]).

Some selected examples of two-dimensional XRD images recorded in parallel to the X-ray absorption measurements employing a photon energy of 9.423 keV (X-ray wavelength λ = 1.3158 Å) are presented in Figure 7. The data collected on the different pixels of the X-ray detector were converted into conventional powder diffractograms and evaluated using the pyFAI software [42]. In comparison to a calculated diffractogram using the structure data of Wurtzite ZnO as already detailed above (PDF file no. 01-079-2205, [38]), it can be concluded that all samples only reveal the expected ZnO diffraction peaks, with the (100) peak at Θ_B_ = 27.0°, the (002) reflection at Θ_B_ = 29.3° and the (101) with Θ_B_ = 30.8°, which implies that the reaction product ZnO can also be detected via the XRD measurements. In agreement with the EXAFS measurements, the presence of ZnO took place with a slight time delay after the initiation of the reaction since a certain volume of crystalline ZnO must first be generated depending on the solution and the temperature. It was only in the reaction of 1-octanol (sample O) that very weak Scherrer rings were detected directly after initiating the reaction (see Figure 7a).

**Figure 8:**
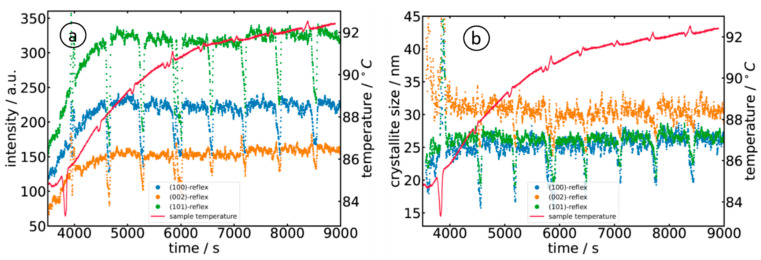
Evaluation of the diffractograms collected during the reaction of Zn(acac)_2_ in 10^−3^ M NaOH (sample A) as a function of time and temperature: (**a**) temporal evolution of the diffraction peak intensities and (**b**) evolution of the crystallite size as calculated by the Scherrer equation. The periodic drops in intensity and crystallite size are due to the periodic interruption of the stirring process, so the particles in the solution tend to move out of the volume probed by the X-ray beam.

For further data analysis of the diffractograms, a polynomial background was subtracted from the diffraction data, followed by a peak fitting with three individual Gaussian peak functions. The evolution of the measured peak intensities of the (100), (002) and (101) reflections is displayed in Figure 8a for sample A, i.e., the reaction of Zn(acac)_2_ in 10^−3^ M NaOH. The three peaks showed an identical trend, i.e., continuously increasing to a maximum intensity at about 6000 s (T ≈ 90 °C). As can already be seen in Figure 7, the detected diffraction peaks exhibited considerable broadening due to the finite length of the X-ray path through the solution containing the nanoparticles, i.e., a particle located closer to the entrance window of the cell shows up at a position further away from the direct beam, while a particle located close to the exit window shows up at a correspondingly shorter distance. Assuming that the particles are homogeneously distributed in the liquid phase, a peak with a flat top can be expected. Such a trend is also experimentally observed by looking at the measured intensity distributions on the flat-panel detector and the extracted diffractogram (Figure 7d). In any case, this broadening must be considered carefully when the peak width is used for an estimation of the crystallite size L, e.g., by employing the Scherrer formula [43]

$$L=\frac{K\;\lambda }{\beta cos\Theta };$$

where λ = 1.3158 Å, β is the full width at half maximum of the considered Bragg peak, Θ is the position of the Bragg peak, and K is the Scherrer constant with a value of 0.9 [43]. The crystallite sizes for the above-mentioned orientations are accordingly determined and plotted in Figure 8b. Similar to the temporal evolution of the intensity, the crystallites grow to a size of about 25 nm ((100) and (101) orientation) and 30 nm ((002)-orientation). The temporal evolution of the diffraction peaks is well in accordance with the presence of small, hollow nanoparticles, which is usually observed in dilute alkaline solutions (see, e.g., refs. [5,10,11]).

In contrast, the use of organic solvents, such as triethanolamine [10], polyvinyl-pyrrolidone [27] or cetyl-trimethyl ammonium bromide [12], lead to needle- or flower-like nanocrystallites with stronger contributions of the otherwise weaker (002) Bragg reflection, and very intense (101) peaks compared to the ZnO reference without a preferred orientation (see the calculation in Figure 7d). This exact behavior was observed in the present study using 1-octanol as a solvent. 

**Figure 9:**
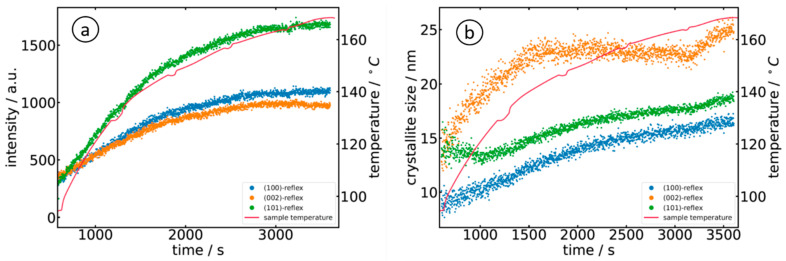
Evaluation of the diffractograms collected during the reaction of Zn(acac)_2_ in the 1-octonal (sample O) as a function of time and temperature: (**a**) temporal evolution of the diffraction peak intensities and (**b**) evolution of the crystallite size as calculated by the Scherrer equation.

Comparing Figure 8a and Figure 9a, we notice the lower intensity of the (002) reflection in the case of the alkali compared to the (100) peak, as well as the dominant (101) reflection and the nearly equal intensities for (100) and (002) for the organic solvent (sample O). It is interesting to note that the intensity (peak area) of the (101) reflection exactly follows the course of the temperature during the reaction, thereby offering an opportunity to manipulate the growth easily. Such behavior is also well in-line with linear growth behavior [44,45]. The evaluation of the crystallite size further shows that the crystallites obey a larger and faster growth along the *c*-axis ((002)-reflection) in the first ca. 1500 s of the reaction, which is in agreement with the presence of needle-like crystallites. The growth of the (100)- and (101)-oriented crystallites was rather slower.

The comparison of the crystallite size found for the two experiments shows that crystallites are slightly larger for the reaction of Zn(acac)_2_ in 10^−3^ M NaOH (sample A), with values of 31 nm ± 2 nm for the (002)-direction and slightly smaller values for (100) and (002) orientations, i.e., 25 nm ± 2 nm after about 4500 s (75 min). When prepared in 1-octanol (sample O), the crystallites were smaller, with a maximum of about 25 nm for (002) and much smaller values for (100) and (101), but with a shorter reaction time of less than one hour. Thus, the particles produced in 1-octanol were significantly smaller than in the NaOH sample, even though identical Zn(acac)_2_ concentrations of the precursor solutions were initially used. The smaller crystallite size in (100) and (101) correlates well with the preparation of hollow nanostructures [11]. Since the diffracted intensity of the diffraction pattern is proportional to the volume leading to the diffraction of the radiation, it can be concluded from the measured Bragg intensities and particle sizes that more, but smaller, nanoparticles are formed in 1-octanol (sample O) than in the alkaline solution (sample A).

Electron microscopy measurements on particles using similar synthesis processes involving, however, a longer synthesis time of several hours or even days showed particle sizes of several hundred nanometers [4,7,11,12]. This indicates that the growth of the ZnO nanoparticles is comprised of the formation of small hexagonal nuclei in the initial stages of growth and a subsequent, temperature-controlled, and eventually non-isotropic growth by diffusion, depending on the solvent. The agglomeration of particles is presumably responsible for the decreased growth rates observed in the alkaline solvent in the later stages of the growth, as all the precursor material in close vicinity of the grown crystallites was already consumed.

### 3.3. Comprehensive Discussion

As described in the sections above, time-resolved quick-scanning EXAFS spectroscopy and X-ray diffraction are able to monitor the formation of hexagonal ZnO nanoparticles during a solvothermal reaction from Zn(acac)_2_. Although EXAFS and XANES are generally very sensitive to small concentrations of additional species of the X-ray absorbing atom, no contributions from the Zn-O intermediate phases could be determined, such as Zn-organometallic species, ZnO-H_2_O, or thermodynamically quite stable tetrameric (ZnO-OH) species, both of which were predicted by density functional calculations (DFT) [10,11]. The situation is similar to the hydrolysis–condensation route of zinc oxy-acetate precursors in ethanolic solution, where only two species were identified by EXAFS and XANES [41]. This could be because the concentrations of these species are beyond the detection limit of the X-ray techniques used, which are on the order of fractions of a percent, or the fact that the short-range order structures of these intermediate complexes are very similar to the hexagonal ZnO structure, with four oxygen atoms in the first coordination sphere (see Table 2). The fact that the amplitude reduction factor S_0_^2^ needed to properly model the first Zn-Zn coordination amounts to values of typically 1.2 and more (see Table 2) may be an indication for an additional Zn-containing phase, as S_0_^2^ is usually approximately 1.0 at its maximum, as it is the case for all the Zn-O coordination spheres. Since the Zn-Zn-coordination number is smaller in the above-mentioned Zn-O complexes, a larger value of S_0_^2^ is needed to compensate for this zinc deficiency in the model fit. Thus, a more detailed data analysis should be performed, including the synthesis, isolation and preparation of suited reference compounds. It should also be mentioned that the hydrolysis of an ethanolic Zn precursor solution with LiOH leads to zinc hydroxide double salts in later stages of the reaction, i.e., after several hours of the reaction [46], so that the presence of additional Zn-O-species would in general not be unexpected. Furthermore, the amorphous Zn-O phases are also reported in the literature (e.g., ref. [47,48]).

Ex situ electron microscopy measurements on the particles derived from similar synthesis processes showed particle sizes of up to several hundred nanometers (e.g., [4,7,9,10]). This leads to the general conclusion that the growth of the ZnO nanoparticles is a sequence of nucleation, diffusion, and subsequent agglomeration and ripening since the measured growth rates decrease rapidly and sharply (see Figure 8 and Figure 9). The growth of the particles could therefore be well described by employing the LaMer model [49]. 

With increasing time and temperature, there is a continuous increase in the ZnO monomer concentration until a critical concentration is reached, and sudden nucleation may occur. This nucleation can be traced by the sharply increasing intensities of the diffraction peaks and the ZnO concentration detected by EXAFS linear combination fits (Figure 6). The subsequent growth of the particles is diffusion-limited; thus, particle growth is substantially faster for an increased reaction temperature. However, due to the better solubility of Zn(acac)_2_ in 1-octanol, the critical concentration is reached more quickly and many Zn nuclei are immediately available for particle formation and growth (sample O). In NaOH and pure water, the critical concentration needed for nucleation is reached with a delay in time, and fewer Zn cations are available for the growth process (samples A and W). Since the nuclei formed only become stable above a certain size, nuclei may dissolve again because there are not enough Zn cations available in the close vicinity of the nucleus to reach the required critical size. This may explain the Bragg peaks observed, almost constant intensities with time. If those small aggregates agglomerate in later stages of growth, the size of the individual crystallites remains more or less unchanged, and only a slight tendency for an Ostwald ripening is observed.

## 4. Conclusions

In this work, the formation of ZnO nanoparticles by hydrothermal synthesis of Zn(acac)_2_ in distilled water and dilute NaOH and solvothermal synthesis in 1-octanol was followed in situ by time-resolved, simultaneous X-ray absorption (EXAFS/XANES) and X-ray diffraction (XRD) measurements, respectively. The simultaneous combination of the two techniques has proven to be very powerful in exploring details of the growth mechanisms of ZnO nanomaterials, as both techniques can investigate different aspects of the reaction. While EXAFS/XANES is suitable for studying both reaction products and reactants as well as possible reaction intermediates with high sensitivity, XRD can provide parallel details about the microstructure of the crystalline reaction products. Depending on the precursor solution and the applied temperature, the formation of hexagonal ZnO could be detected in all solvents by EXAFS and XRD and most effectively in 1-octanol with a conversion of more than 80% after about 35 min. The prepared nanoparticles showed preferential growth along the c-axis of the hexagonal lattice structure, which is most pronounced in 1-octanol with needle-like deposits.

The growth of the nanoparticles, in particular the observed anisotropy, can be explained using the LaMer model. After a critical ZnO cation concentration has been reached, there is a sudden, instantaneous nucleation with subsequent diffusion-controlled growth. Consequently, the solution is depleted in the Zn(acac)_2_-precursor in the vicinity of the formed ZnO nuclei. Thus, stable nuclei may build up smaller aggregates, so the final particle size is determined by agglomeration and Ostwald ripening. The different degrees of solubility of Zn(acac)_2_ in the various solvents used here obviously affect the ZnO growth processes. Zn(acac)_2_ dissolves most readily in 1-octanol, which is why the critical concentration is already reached at room temperature and a large number of ZnO cations are available for particle growth, i.e., many small nuclei are formed compared to the dilute alkali (sample A) or distilled water (sample W) as solvents. The results of the present study allow us to easily determine the preparation conditions for tailored nanoparticles in an in situ experiment. By varying the solvent, the reaction temperature, the pressure in the cell, and the reaction time, particles with the desired shape, size, and crystallographic structure can be obtained.

For future investigations, it might be useful to additionally couple UV–Vis spectroscopy [18,41,50,51], Raman spectroscopy [50,52], small angle X-ray scattering [41,53] or FT-IR [54] to the combined X-ray measurements to obtain additional physicochemical properties of the formed particles and deeper insights into the reaction mechanisms and dynamics.

Furthermore, the present setup only allows monitoring a rather small angular range of the X-ray diffraction pattern. A larger detector with smaller pixels would facilitate measurements of more diffraction peaks and thus provide a more detailed insight into the microstructure of the grown particles. Regarding the detection of intermediate reaction products, the present experiments suggest that the reaction from the Zn(acac)_2_ precursor to the ZnO occurs directly, i.e., without reaction intermediates. Quick-EXAFS with a faster repetition rate should be performed to exclude the presence of short-living intermediates. At present, the technical limitations of the method are around 5-10 ms for an individual spectrum [32,55], so the transient species with a subsecond lifetime should be feasible to detect.

## Figures and Tables

**Table 1:** Compilation of the sample preparations and sample identification discussed in this work.

Sample Id.	Zn(acac)_2_H_2_O	Solvent	Amount	Temperature	Time
A	0.75 g	10^−3^ M NaOH	50 ml	90 °C	150 min
O	0.75 g	1-octanol	50 ml	165 °C	60 min
W	0.75 g	water	50 ml	90 °C	450 min

**Table 2:** Compilation of quantitative fit results obtained from a detailed EXAFS data analysis for samples A, O and W after extraction of the synthesized ZnO nanomaterials from the reaction cell.

Sample	Shell	N	S_0_^2^	R/Å	σ^2^/Å^2^	E_0_/eV
Sample A (R-factor: 0.025)	Zn-O	4	0.793 ± 0.097	1.978 ± 0.007	0.0051 ± 0.0012	2.65 ± 0.69
	Zn-Zn	12	1.313 ± 0.210	3.247 ± 0.006	0.0122 ± 0.0012	2.65 ± 0.69
	Zn-O	1	0.793 ± 0.097	3.255 ± 0.007	0.0051 ± 0.0012	2.65 ± 0.69
	Zn-O-O	12	0.793 ± 0.097	3.600 ± 0.008	0.0051 ± 0.0012	2.65 ± 0.69
	Zn-O-Zn	24	1.053 ± 0.155	3.600 ± 0.008	0.0087 ± 0.0012	2.65 ± 0.69
	Zn-O	9	0.793 ± 0.097	3.808 ± 0.008	0.0051 ± 0.0012	2.65 ± 0.69
	Zn-O	4	0.793 ± 0.097	3.964 ± 0.009	0.0051 ± 0.0012	2.65 ± 0.69
	Zn-O-Zn-O	12	1.053 ± 0.155	3.964 ± 0.009	0.0087 ± 0.0012	2.65 ± 0.69
	Zn-O-O	6	0.793 ± 0.097	4.229 ± 0.009	0.0051 ± 0.0012	2.65 ± 0.69
	Zn-O-Zn	36	1.053 ± 0.155	4.516 ± 0.009	0.0087 ± 0.0012	2.65 ± 0.69
	Zn-O-O	36	0.793 ± 0.097	4.516 ± 0.009	0.0051 ± 0.0012	2.65 ± 0.69
	Zn-Zn-O	36	1.053 ± 0.155	4.516 ± 0.009	0.0087 ± 0.0012	2.65 ± 0.69
	Zn-Zn	6	1.313 ± 0.210	4.577 ± 0.010	0.0122 ± 0.0012	2.65 ± 0.69
	Zn-O	6	0.793 ± 0.097	4.599 ± 0.010	0.0051 ± 0.0012	2.65 ± 0.69
Sample O (R-factor: 0.030)	Zn-O	4	0.854 ± 0.114	1.971 ± 0.008	0.0047 ± 0.0012	3.12 ± 0.75
	Zn-Zn	12	1.573 ± 0.227	3.245 ± 0.006	0.0126 ± 0.0013	3.12 ± 0.75
	Zn-O	1	0.854 ± 0.114	3.250 ± 0.007	0.0047 ± 0.0012	3.12 ± 0.75
	Zn-O-O	12	0.854 ± 0.114	3.595 ± 0.008	0.0047 ± 0.0012	3.12 ± 0.75
	Zn-O-Zn	24	1.214 ± 0.096	3.595 ± 0.008	0.0087 ± 0.0013	3.12 ± 0.75
	Zn-O	9	0.854 ± 0.114	3.802 ± 0.009	0.0047 ± 0.0012	3.12 ± 0.75
	Zn-O	4	0.854 ± 0.114	3.958 ± 0.009	0.0047 ± 0.0012	3.12 ± 0.75
	Zn-O-Zn-O	12	1.214 ± 0.096	3.958 ± 0.009	0.0087 ± 0.0013	3.12 ± 0.75
	Zn-O-O	6	0.854 ± 0.114	4.223 ± 0.009	0.0047 ± 0.0012	3.12 ± 0.75
	Zn-O-Zn	36	1.214 ± 0.096	4.509 ± 0.010	0.0087 ± 0.0013	3.12 ± 0.75
	Zn-O-O	36	0.854 ± 0.114	4.509 ± 0.010	0.0047 ± 0.0012	3.12 ± 0.75
	Zn-Zn-O	36	1.214 ± 0.096	4.509 ± 0.010	0.0087 ± 0.0013	3.12 ± 0.75
	Zn-Zn	6	1.573 ± 0.277	4.570 ± 0.010	0.0126 ± 0.0013	3.12 ± 0.75
	Zn-O	6	0.854 ± 0.114	4.592 ± 0.010	0.0047 ± 0.0012	3.12 ± 0.75
Sample W (R-factor: 0.029)	Zn-O	4	0.735 ± 0.096	1.972 ± 0.008	0.0048 ± 0.0012	3.62 ± 0.74
	Zn-Zn	12	1.258 ± 0.222	3.244 ± 0.006	0.0123 ± 0.0013	3.62 ± 0.74
	Zn-O	1	0.735 ± 0.096	3.248 ± 0.007	0.0048 ± 0.0012	3.62 ± 0.74
	Zn-O-O	12	0.735 ± 0.096	3.593 ± 0.008	0.0048 ± 0.0012	3.62 ± 0.74
	Zn-O-Zn	24	0.997 ± 0.112	3.593 ± 0.008	0.0086 ± 0.0013	3.62 ± 0.74
	Zn-O	9	0.735 ± 0.096	3.800 ± 0.009	0.0048 ± 0.0012	3.62 ± 0.74
	Zn-O	4	0.735 ± 0.096	3.956 ± 0.009	0.0048 ± 0.0012	3.62 ± 0.74
	Zn-O-Zn-O	12	0.997 ±0.112	3.956 ± 0.009	0.0086 ± 0.0013	3.62 ± 0.74
	Zn-O-O	6	0.735 ± 0.096	4.220 ± 0.009	0.0048 ± 0.0012	3.62 ± 0.74
	Zn-O-Zn	36	0.997 ± 0.112	4.506 ± 0.010	0.0086 ± 0.0013	3.62 ± 0.74
	Zn-O-O	36	0.735 ± 0.096	4.506 ± 0.010	0.0048 ± 0.0012	3.62 ± 0.74
	Zn-Zn-O	36	0.997 ± 0.112	4.506 ± 0.010	0.0086 ± 0.0013	3.62 ± 0.74
	Zn-Zn	6	1.258 ± 0.222	4.567 ± 0.010	0.0123 ± 0.0013	3.62 ± 0.74
	Zn-O	6	0.735 ± 0.096	4.589 ± 0.010	0.0048 ± 0.0012	3.62 ± 0.74

## Data Availability

The data presented in this manuscript will be available from the authors upon reasonable request.
